# Computational Approaches for Cancer-Fighting: From Gene Expression to Functional Foods

**DOI:** 10.3390/cancers13164207

**Published:** 2021-08-21

**Authors:** Francesco Monticolo, Maria Luisa Chiusano

**Affiliations:** Department of Agricultural Sciences, Università degli Studi di Napoli Federico II, Via Università 100, 80055 Portici, Italy; francesco.monticolo@unina.it

**Keywords:** bioactive compounds, apoptosis, programmed cell death, bioinformatics, gene expression, survival analysis

## Abstract

**Simple Summary:**

It is today widely accepted that a healthy diet can be one of the fundamental approaches to prevent the risk of cancer. To this aim, nutrigenomics studies are indeed providing a precious source of information, favoring the search for compounds that could affect gene expression in a favorable way. Here we present a computational study to select candidate compounds that could play a role in cancer prevention and care. Starting from analyses of gene expression, we identified 7 genes that have opposite expression trends in apoptotic treatments when compared with 8 different cancer types. In addition, based on structure similarity with 6 compounds that affect the expression patterns of these genes in a favorable way against 8 cancer types, we selected 23 natural compounds as suitable candidates for further tests as possible novel drugs or for the design of functional food for cancer treatment and prevention.

**Abstract:**

It is today widely accepted that a healthy diet is very useful to prevent the risk for cancer or its deleterious effects. Nutrigenomics studies are therefore taking place with the aim to test the effects of nutrients at molecular level and contribute to the search for anti-cancer treatments. These efforts are expanding the precious source of information necessary for the selection of natural compounds useful for the design of novel drugs or functional foods. Here we present a computational study to select new candidate compounds that could play a role in cancer prevention and care. Starting from a dataset of genes that are co-expressed in programmed cell death experiments, we investigated on nutrigenomics treatments inducing apoptosis, and searched for compounds that determine the same expression pattern. Subsequently, we selected cancer types where the genes showed an opposite expression pattern and we confirmed that the apoptotic/nutrigenomics expression trend had a significant positive survival in cancer-affected patients. Furthermore, we considered the functional interactors of the genes as defined by public protein-protein interaction data, and inferred on their involvement in cancers and/or in programmed cell death. We identified 7 genes and, from available nutrigenomics experiments, 6 compounds effective on their expression. These 6 compounds were exploited to identify, by ligand-based virtual screening, additional molecules with similar structure. We checked for ADME criteria and selected 23 natural compounds representing suitable candidates for further testing their efficacy in apoptosis induction. Due to their presence in natural resources, novel drugs and/or the design of functional foods are conceivable from the presented results.

## 1. Introduction

In 2021, in the United States, 1,898,160 new cancer cases and 608,570 cancer deaths are expected [1]. Diagnosis of cancer affects not only patients with physical sufferings and possibility of death, but also reduces the quality of life for their family or caregivers [2]. In 2018, the total cost of cancer care was around €103 billion, of which €32 billion were spent on cancer drugs in Europe [3]. In US, the overall national costs in 2015 were $183 billion and projected to become $246 billion in 2030. The overall annualized average costs were highest in the final stage of cancer, followed by the initial and continuing phases (medical care: $105,500, $41,800, $5300 and oral prescription drugs: $4200, $1800, $1100, respectively) [4]. These are the main reasons that make the research on cancer prevention pivotal for healthcare and the whole society as well.

Cancer hallmarks include proliferative signaling, elusion of programmed cell death, evasion to immune destruction in favor of replicative immortality, angiogenesis, metastasis, and reprogramming of energy metabolism [5]. The genome instability is the main factor triggering the acquisition of these hallmarks, also generating genetic diversity [5]. The “-omics” explosion consistently improved the knowledge on cancer genetics and functional diversity as well as the number of data resources to be exploited for the research in the field. Among these resources, the Catalogue Of Somatic Mutations In Cancer (COSMIC) is the most detailed and comprehensive resource for exploring the effect of somatic mutations in human cancer [6]. The latest release, COSMIC v94 (May 2021), includes almost 38 million coding mutations across 1.5 million tumor samples, curated from over 28,000 publications [6].

The International Cancer Genome Consortium (ICGC) is a global initiative to build a comprehensive catalog of mutational abnormalities encompassing 90 different cancer projects [7]. Eighty-six out of these 90 projects are available through the ICGC portal, spanning 22 primary cancer sites (Release 28), providing genomic, transcriptomic, and epigenomic datasets of specific cancer types [7].

The Cancer Genome Atlas (TCGA) project provides large-scale multi-dimensional analyses of multiple chromosomal aberrations, nucleotide substitutions and epigenetic modifications that drive malignant transformations [8]. In 2014, the TCGA Research Network reported on 3527 tumors from 12 different cancer types, integrating different -omics platforms to assay tumor DNA, RNA and a cancer-relevant set of proteins and phosphoproteins [9]. Currently, TCGA provides data from 33 different tumor types (the PanCancer Atlas), ranging from genomics and epigenomics to transcriptomics and proteomics. These data can help to address still unanswered intriguing questions [10], paving the way to novel opportunities in cancer research.

Interestingly, in 2020 [11], an international collaboration between ICGC and TCGA, called Pan-Cancer Analysis of Whole Genomes Consortium (PCAWG), examined the features and possible consequences of genomic variations by focusing on both coding and non-coding regions of 2658 whole-cancer genomes [11].

Today, it is widely accepted that cancer risk avoidance as well as health and care, may reside on a proper lifestyle, in which the diet has a fundamental role, in terms of weight control, physical activity and food quality [2]. A healthy eating pattern is mainly based on foods that have balanced amounts of good quality nutrients (vegetables, fiber-rich legumes, fruits and whole grains), and on limited amounts of red and processed meats, sugar-sweetened beverages, highly processed foods and refined grain products [2]. In addition, specific bioactive compounds, such as sterols, indoles and phenols, naturally present in many foods, mainly in vegetables and fruits, are known to enrich food quality [12], acting as protective, preventing and caring agents in inflammation [13,14], disease [15,16], infections [17,18] and in chronic diseases such as cancer [19,20]. Indeed, it has been shown that improved diet quality reduces the risk of cancer deaths from 11% to 23% [21].

The increasing attention towards a proper exploitation of natural compounds also determined the increasing amount of nutrigenomics experiments and associated resources. Among these, the Monarch Initiative [22] and the Comparative Toxicogenomics Database (CTD) [23] denote relationships between nutrients, disease, phenotypes and genes [24]. The Monarch Initiative integrates information on genes, genotypes, phenotypes and diseases in a variety of species, and allows powerful ontology-based search [22]. CTD is a database that relates toxicological information for chemicals, genes, phenotypes, diseases, and exposures, to advance the understanding about human health [23]. CTD included over 2.7 million manually curated data from interactions of chemical–gene, chemical–phenotype, chemical–disease, gene–disease and chemical–exposure [23].

FooDB (http://www.foodb.ca, accessed on 13 May 2021), contains information on more than 28,000 chemicals found in more than 1000 unprocessed food products.

NutriGenomeDB [25], is based on a set of manually curated differentially expressed genes, obtained using human cell-based assays from microarray, after treatment with nutrients or bioactive food compounds [25].

Based on the appropriate exploitation of currently available bioinformatics resources, we recently identified 734 genes that resulted co-expressed with genes involved in programmed cell death but not reported as implicated in this process by gene ontology (GO) or Kyoto Encyclopedia of Genes and Genomes (KEGG) pathways [26]. We further investigated this collection with the principal aim to identify those genes that have the same expression pattern between apoptotic previous results and nutrigenomics treatments inducing cell death [25]. We selected 149 genes from a total of 24 apoptosis-inducing treatments (15 from nutrigenomics experiments) that were further analyzed in cancer treatments, to identify cancer types in which they showed opposite expression trends [27]. We identified 22 genes that were further investigated for the correlation of their expression variability and patient survival [28]. We identified a subset of 7 candidate genes that are associated to a favorable outcome considering nutrigenomics treatments with 6 compounds. The function interactors of the 7 encoded proteins confirmed their role in apoptosis and/or cancer [29]. Further investigations based on the similarity [30] with 5 out of 6 compounds lead to the identification of 231 similar compounds among which 23 confirmed the adsorption, the distribution, the metabolism and the excretion (ADME) parameters [31]. These 23 bioactive compounds are here proposed for further testing for their role in preventing or treating the cancer types here considered. Finally, using FooDB (http://www.foodb.ca) (accessed on 13 May 2021), we identified foods that contain 4 out of the 23 compounds that resulted potentially useful for the prevention of non-Hodgkin lymphoma and skin melanoma.

## 2. Materials and Methods

We here present a deeper investigation on the 734 genes from [26]. These genes are co-expressed in 9 apoptotic treatments (Tamoxifen (TMX) [32], Zika virus (NCC-Zika and HNP-Zika) [33], Vitamin C (Vitamin C) [34], ethanol (Ethanol) [35], a CBFβ-SMMHC inhibitor (Al-10-49) [36], Pam3CSK4 (PAM3h and PAM24h) [37] and a KDM1A inhibitor (NCD38) [38]), together with genes involved in programmed cell death. However, the 734 genes in the dataset were not previously reported to be implicated in programmed cell death according to the GOs or KEGG pathway annotations [39].

We investigated the expression of these genes in 231 manually curated results from nutrigenomics human cell-based treatments (treatments with nutrients or bioactive food compounds) analyzed by the Affymetrix microarray, and available from the NutriGenomeDB [25]. Only the 10% of the most differentially expressed genes are included in the database [25]. Among these, we considered the nutrigenomics treatments that are known to induce apoptosis [34,35,36,37,38,39,40,41,42,43,44,45,46,47,48], and considered those genes with a False Discovery Ratio (FDR) < 0.05 and a |log2 Fold Change| > 1.5. The comparison of the gene expression patterns in apoptosis determining nutrigenomics treatments with those of the 9 independent apoptotic treatments from [26] resulted in a list of 149 differentially expressed genes showing the same trend (up- or down-regulation) in the two classes of apoptotic treatments.

The 149 genes were further investigated for their expression in cancer using GEPIA2 [27]. GEPIA2 is a web server for expression profiling of data in TCGA [8], The Cancer Genome Atlas, and in the Genotype-Tissue Expression (GTEx) [49]. The differential expression per gene in each cancer type was determined using LIMMA option and filtering an FDR < 0.01 and a |log2 Fold Change| > 1. We considered only the genes differentially expressed in at least one cancer, and, in one case, that had a uniform behavior (up- or down-regulation) in different cancers in which they resulted differentially expressed, and, in both cases, we considered only those showing an opposite expression pattern when compared with those from apoptotic treatments (i.e., those up-regulated in apoptotic treatments and down-regulated in at least one cancer type or those down-regulated in apoptotic treatments and up-regulated in at least one cancer type). This filtering resulted in a list of 22 genes that have a distinct behavior in cancers and in apoptotic treatments, thus revealing potential candidates as prognostic markers. The survival analysis of the 22 genes was performed using UALCAN [28], that provides the plots and the statistics concerning the survival analysis associated to gene expression trends in the different cancers [28]. Seven genes showed an enhanced survival plot (*p*-value < 0.05) when the expression pattern was similar to the one shown in apoptotic-induced treatments, and opposite to cancer.

Protein-protein interaction analysis on the 7 genes was performed using STRING [29] that collects and integrates all publicly available sources of protein-protein interaction data [29]. Only interaction with 0.500 “minimum required interaction score” and 25 “max number of interactors to show” were considered.

To identify additional bioactive natural compounds that could induce similar effects to those caused by the compounds already exploited in the nutrigenomics treatments, we used SwissSimilarity [30], that allows to perform a ligand-based virtual screening of libraries of small molecules. The working hypothesis of the virtual screening is that similar molecules could determine similar biological activity [30]. The SMILE format of the 5 initial compounds was obtained using PubChem [50]. The screening was performed using the combined score considering bioactive compounds from all libraries: PDB [51], ChEMBL (activity <10 µM) [52], ChEBI [53], kinase inhibitors (ChEMBL) [52], GPCR Ligands (ChEMBL) [52], GPCR ligands (GLASS) [54] and human metabolomic database (HMDB) [55]. Only compounds showing a similarity score >0.6 with the 5 references were considered, since this is suggested to be the lower threshold to expect similar activity based on structure similarity relationships [56]. The resulting bioactive compounds were also analyzed for their pharmacokinetics, drug likeness and medicinal chemistry friendliness using SwissADME [31], to compute the physicochemical descriptors and predict the adsorption, the distribution, the metabolism and the excretion (ADME) parameters of small molecules to support drug discovery [31]. Only bioactive compounds with 0 violation of the Lipinski [57], Ghose [58], Veber [59], Egan [60] and Muegge [61] methods and ≤1 violation in Pan Assay Interference Compounds (PAINS) [62], Brenk [63], lead likeness [64] and high gastrointestinal absorption were considered.

Possible unprocessed foods possibly containing one of the bioactive compounds were investigated in FooDB (http://www.foodb.ca, accessed on 13 May 2021), which contains information on more than 28,000 chemicals found in more than 1000 unprocessed food products. The data in FooDB are collected from textbooks, scientific journals, online food composition or nutrient databases, flavor and aroma databases (http://www.foodb.ca, accessed on 13 May 2021).

## 3. Results

### 3.1. Programmed Cell Death Candidate Genes and Their Response in Apoptosis-Inducing Nutrigenomics Treatments

The 734 genes that resulted co-expressed with genes involved in programmed cell death in 9 apoptotic treatments [26] were investigated exploiting NutriGenomeDB [25]. This analysis resulted in 149 genes that showed the same expression pattern in 15 nutrigenomics experiments inducing apoptosis (Table S1).

### 3.2. Search for Gene Involvement in Cancer

**Figure 1:**
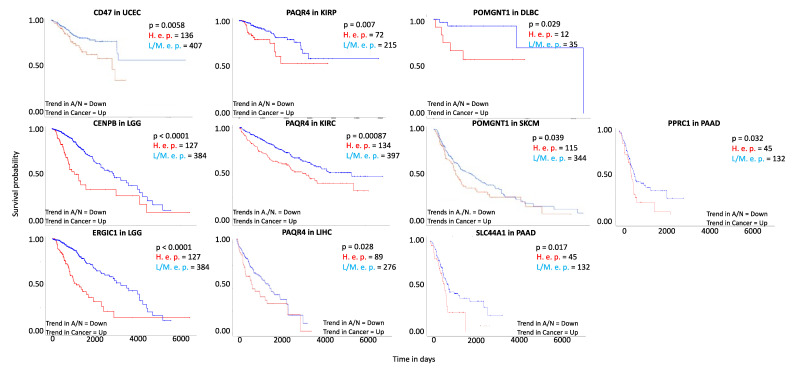
Survival plots (Kaplan–Meier curves) displaying effects of the 7 candidate genes from clinical studies. On the y-axis, the survival probability, and on the x-axis, the time (in days) reported for each considered gene are shown. H. e. p. (High expression patients) and L/M. e. p. (Low/Medium expression patients) indicate the number of patients showing a gene expression value > 3rd quartile and ≤ 3rd quartile, respectively, for each candidate gene. The statistical significance of the difference between the survival curves of H. e. p. and L/M. e. p is p (*p*-value by the log-rank test). Trends in apoptotic, nutrigenomic treatments (Trend in A/N) and in cancer (Trend in Cancer) of gene expression (Up- or Down-regulated) are also reported. The plots suggest more favorable outcomes in patients showing the expression trends corresponding to those reported for apoptotic and nutrigenomic treatments. Note: DLBC: lymphoid neoplasm diffuse large B-cell lymphoma, KIRC: kidney renal clear cell carcinoma, KIRP: kidney renal papillary cell carcinoma, LGG: brain lower-grade glioma, LIHC: liver hepatocellular carcinoma, PAAD: pancreatic adenocarcinoma, SKCM: skin cutaneous melanoma, UCEC: uterine corpus endometrial carcinoma.

The cross comparison between the gene expression of the 149 genes responsive to apoptosis and their behavior in 33 different tumor types reported in GEPIA2 [27], permitted to select 22 genes that showed a significant differential expression in cancer, while being not expressed or with opposite trends in the apoptotic/nutrigenomics treatments (Table S2). We therefore checked for the survival analysis of the specific gene using UALCAN [28] in the cancer types confirming this behavior. Seven genes in 9 different conditions, corresponding to 8 different cancer types, showed an enhanced survival profile when considering similar trends as in the apoptotic/nutrigenomics treatments, suggesting their pro-apoptotic role and, therefore, their possible involvement in cancer studies (Table 1, Figure 1).

*CD47* is down-regulated in Tamoxifen apoptotic treatment, in Withaferin A and Indole-3-Carbinol nutrigenomics treatments, while it is up-regulated in 11 different cancer types. The survival analysis in uterine corpus endometrial carcinoma demonstrates a higher survival probability of individuals with medium/low expression levels of *CD47*, than individuals with high expression levels of this gene (Figure 1, Table 1).

The gene *CENPB* is down-regulated in Al-10-49 apoptotic treatment, in Withaferin A and Indole-3-Carbinol nutrigenomics treatments, while it is up-regulated in 5 different cancer types. The survival analysis in brain lower-grade glioma demonstrates a higher survival probability of individuals with medium/low expression level of *CENPB* than in individuals with high expression levels (Figure 1, Table 1).

The gene *ERGIC1* is down-regulated under Tamoxifen apoptotic treatment and in Indole-3-Carbinol nutrigenomics treatment, while it is up-regulated in 7 different cancer types. The survival analysis in brain lower-grade glioma demonstrates a higher survival probability of individuals with medium/low expression level of *ERGIC1*, than in individuals with high expression levels (Figure 1, Table 1).

The gene *PAQR4* is down-regulated under Al-10-49 and Tamoxifen apoptotic treatments, rosemary, Withaferin A and Eusynstyelamide B nutrigenomics treatments, while it is up-regulated in 21 different cancer types. The survival analysis in kidney renal clear cell carcinoma, kidney renal papillary cell carcinoma and liver hepatocellular carcinoma demonstrates a high survival probability of individuals with medium/low expression level of *PAQR4* than in individuals with high expression levels (Figure 1, Table 1).

The gene *POMGNT1* is down-regulated under Al-10-49 apoptotic treatment and in Bruceine D nutrigenomics treatment, while it is up-regulated in 4 different cancer types. The survival analysis shows a high survival probability in lymphoid neoplasm diffuse large B-cell lymphoma and skin cutaneous melanoma of individuals with medium/low expression level of *POMGNT1* than in individuals with high expression levels (Figure 1, Table 1).

The gene *PPRC1* is down-regulated under Al-10-49 apoptotic treatment, Indole-3-Carbinol and Japonicone A nutrigenomics treatments, while it is up-regulated in 3 different cancer types. The survival analysis in pancreatic adenocarcinoma demonstrates a high survival probability of individuals with medium/low expression level of *PPRC1* than in individuals with high expression levels (Figure 1, Table 1).

Finally, the gene *SLC44A1* is down-regulated under Tamoxifen apoptotic treatment, Indole-3-Carbinol and Withaferin A nutrigenomics treatments, while it is up-regulated in 12 different types of cancers. The survival analysis in pancreatic adenocarcinoma demonstrates a high survival probability of individuals with medium/low expression level of *SLC44A1* than in individuals with high expression levels (Figure 1, Table 1).

### 3.3. Protein-Protein Interaction Patterns to Infer on Gene Product Functionality

In order to investigate the possible interactions of the proteins encoded by the 7 genes under analysis, we used the STRING database [29].

**Figure 2:**
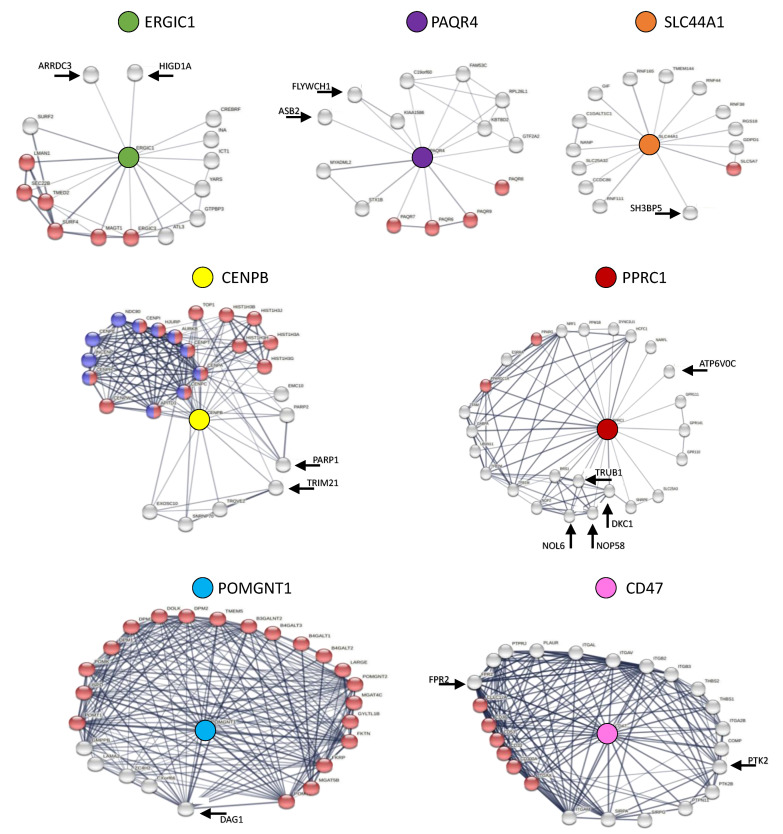
Functional interactions of the 7 candidate genes revealed by the STRING analysis. CENPB (yellow ball) mainly interacts with proteins involved in chromatin organization (red balls), and proteins involved in centromere formation (blue balls). ERGIC1 (green ball) mainly interacts with proteins involved in vesicle-mediated transport (red balls). PAQR4 (purple ball) mainly interacts with other adipoQ receptor (red balls). POMGNT1 (light-blue ball) mainly interacts with proteins involved in glycosylation (red balls). PPRC1 (brown ball) interacts mainly with other peroxisome proliferator-activated receptors (red balls). SCL44A1 (orange ball) interacts mainly with other choline transporters (red balls). CD47 (pink ball) interacts mainly with other CD proteins (red balls). Black arrows and conventional gene names indicate other functional interactors with documented involvement in cancer or programmed cell death.

The gene *CD47* encodes for a membrane receptor that belongs to the cluster of differentiation proteins of the immunoglobulin superfamily [65]. Investigating on possible interacting proteins from STRING (Figure 2), CD47 interacts with other clusters of differentiation proteins, and also with a tyrosine kinase (PTK2), a cytoplasmic protein that is known to be cleaved by caspase 3 during cell death [66], interrupting survival signals from the extracellular matrix, and a G-protein coupled receptor (FPR2) that is reported to have an anti-apoptotic function in colorectal cancer [67].

*CENPB* encodes for the major centromere autoantigen B that facilitates centromere formation [68]. Interestingly, investigating on CENPB protein interactors (Figure 2), the results associated not only with other proteins involved in centromere formation, but also with PARP1 which is known to be involved in programmed cell deaths [69,70,71], in cancer [72], and in the cellular response to DNA damage [73]. CENPB interacts also with TRIM21, an E3 ubiquitin ligase, involved in innate immunity, associated to cancer proliferation, as well as in systemic lupus erythematosus and in Sjögren’s syndrome [74].

*ERGIC1* encodes for a protein that is involved in the transport from the endoplasmic reticulum to the Golgi apparatus [75], as reported by the STRING data (Figure 2). In addition, the protein-protein interaction pattern also shows that ERGIC1 interacts with a member of the arrestin protein family (ARRDC3), which is proposed to be a breast cancer tumor suppressor [76], and with HIGD1A, which resides in mitochondria during physiological condition, and is known to be accumulated in the nuclei during severe metabolic stress or upon DNA damage [77].

The gene *PAQR4* encodes for a progestin and adipoQ receptor [78]. Interestingly, PAQR4 results to interact with an ankyrin repeat protein (ASB2) that is involved in chromatin condensation [79], and with a suppressor of the nuclear β-catenin (FLYWCH1) [80], that inhibits cell migration and metastasis formation (Figure 2).

The gene *POMGNT1* encodes for an O-mannose beta-1,2-*N*-acetylglucosaminyltransferase which participates in O-mannosyl glycan synthesis [81,82]. POMGNT1 not only interacts with other transferases that are involved in the biosynthesis of the phosphorylated saccharides, but also with the dystroglycan (DAG1) protein, involved in extracellular matrix organization. Related defects separate the epithelial and stromal compartments, which is considered a hallmark of malignant transformation [83] and in dystrophic phenotypes [84] (Figure 2).

*PPRC1* encodes for a peroxisome proliferator-activated receptor γ coactivator 1 which is linked to mitochondrial biogenesis [85]. It interacts with other peroxisome proliferator-activated receptor coactivators, and also with proteins involved in ribosome biogenesis (DKC1, NOL6 and NOP56), which are known to be associated with ribosomal dysfunction and increased cancer susceptibility in the human X-linked dyskeratosis congenital [86,87]. PPRC1 also interacts with a protein involved in mRNA pseudouridylation (TRUB1) and with a vacuolar ATPase (ATP6V0C), which is known to be involved in the maintenance of pH homeostasis [88] (Figure 2).

The gene *SLC44A1* encodes for a mediator of the choline transport across both the plasma and the mitochondrial membranes [89]. It interacts with other choline transporters but also with a guanine nucleotide exchange factor (SH3BP5). Interestingly, the knockdown of SH3BP5 is known to induce apoptosis in leukemia cells [90] (Figure 2).

### 3.4. Nutrients and Bioactive Compounds

As reported in Table 1, the 7 genes here considered show the same expression trends when investigated in nutrigenomics treatments based on 6 natural compounds determining apoptosis (Table 1). Interestingly, all the genes are down-regulated in apoptotic treatments with enhanced survival plot when low/medium expressed in patients with cancer. The rosemary extract affects *PAQR4* expression that when low/medium expressed, affects the survival in patients with kidney renal clear cell carcinoma, kidney renal papillary cell carcinoma and liver hepatocellular carcinoma (Table 1). Indole-3-carbinol affects the expression of 5 different genes (*CD47*, *CENPB*, *ERGIC1*, *PPRC1*, *SLC44A1*) that, in turn, affect the survival in patients with uterine corpus endometrial carcinoma (*CD47*), brain lower-grade glioma (*CENPB, ERGIC1*) and pancreatic adenocarcinoma (*PPRC1, SLC44A1*), respectively (Table 1). Bruceine D affects *POMGNT1* expression that, in turn, affects the survival in patients with lymphoid neoplasm diffuse large B-cell lymphoma and skin cutaneous melanoma (Table 1). Withaferin A affects the expression of 4 different genes (*CD47*, *CENPB*, *PAQR4*, *SLC44A1*) that, in turn, affect the survival plots in patients with uterine corpus endometrial carcinoma (*CD47*), brain lower-grade glioma (*CENPB*), kidney renal clear cell carcinoma (*PAQR4*), kidney renal papillary cell carcinoma (*PAQR4*), liver hepatocellular carcinoma (*PAQR4*) and pancreatic adenocarcinoma (SLC44A1) (Table 1). Japonicone A affects *PPRC1* expression that, in turn, affects the survival in patients with pancreatic adenocarcinoma (Table 1). Eusynstyelamide B affects *PAQR4* expression that, in turn, affects the survival in patients with kidney renal clear cell carcinoma, kidney renal papillary cell carcinoma and liver hepatocellular carcinoma (Table 1).

**Figure 3:**
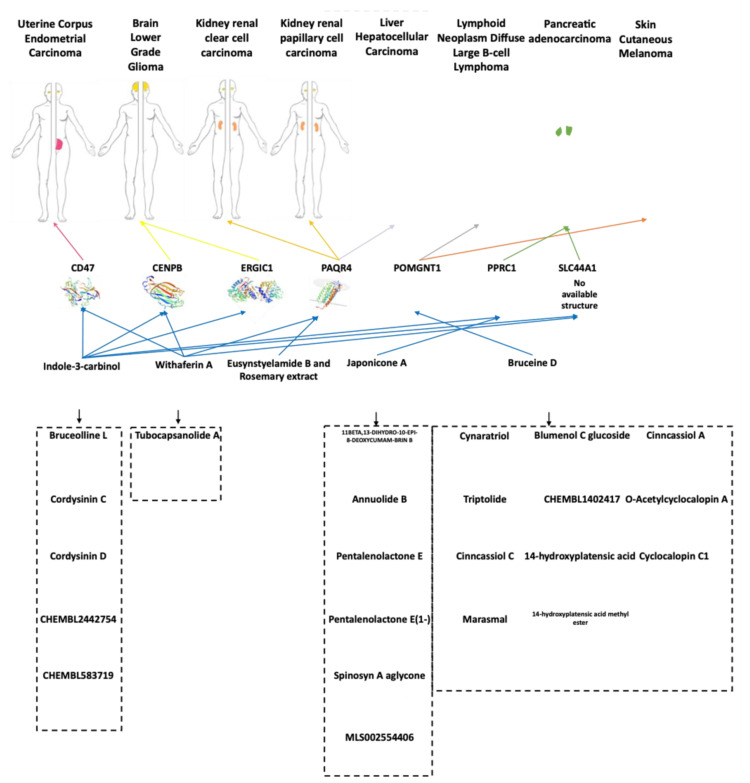
Natural compounds proposed for treatments in specific cancers. The 5 reference compounds (Bruceine D, Eusynstyelamide B, Japonicone A, Withaferin A and Indole-3-carbinol) associated to the specific cancer types where they can have potential efficacy, and the corresponding similar ones (enclosed in dashed rectangles) detected by SwissSimilarity and SwissADME (23 in total) are shown. Five compounds are similar to Indole-3-Carbinol, which triggers the down-regulation of 5 different genes. In particular, the down-regulation of *CD47* enhances the survival in uterine corpus endometrial carcinoma; the down-regulation of *CENPB* and *ERGIC1* enhances the survival in brain lower-grade glioma; the down-regulation of *PPRC1* and *SLC44A1* enhances the survival in pancreatic adenocarcinoma. Eleven compounds are similar to Bruceine D, that triggers the down-regulation of *POMGNT1* that enhances the survival in lymphoid neoplasm diffuse large B-cell lymphoma and skin cutaneous melanoma. One compound, that is similar to Withaferin A, triggers the down-regulation of 4 different genes. In particular, the down-regulation of *CD47* enhances the survival in uterine corpus endometrial carcinoma, the down-regulation of *CENPB* enhances the survival in brain lower-grade glioma; the down-regulation of *PAQR4* enhances the survival in kidney renal clear cell carcinoma, in kidney renal papillary cell carcinoma and in liver hepatocellular carcinoma, and the down-regulation of *SLC44A1* enhances the survival in pancreatic adenocarcinoma. Six compounds are similar to Japonicone A, that triggers the down-regulation of *PPRC1*, that enhances the survival in pancreatic adenocarcinoma.

The five reference natural compounds (with the exception of the rosemary extract that does not have a defined molecular organization) were used in SwissSimilarity [30] to identify similar chemical compounds. The working hypothesis behind SwissSimilarity is that similar molecules are prone to exhibit similar biological activity [30]. We found 231 compounds with similarity to at least one of the reference compounds (Table S3). SwissADME [31] was used to classify the physicochemical descriptors and to predict the human adsorption, distribution, metabolism and excretion (ADME) of the compounds. Twenty-three bioactive compounds with 0 violation to the Lipinski [57], Ghose [58], Veber [59], Egan [60] and Muegge [61] methods; ≤1 violation in Pan Assay Interference Compounds (PAINS) [62], Brenk [63], lead likeness [64] and with high gastrointestinal absorption were selected (Table S4), and these were reported in association with their chemical reference (Figure 3).

In particular, 5 compounds are similar to Indole-3-Carbinol, thus representing additional compounds to be exploited and tested for their possible induction of the down-regulation of the 5 different genes (*CD47, CENPB, ERGIC1, PPRC1, SLC44A1*), that resulted to enhance the survival in uterine corpus endometrial carcinoma (*CD47*), brain lower-grade glioma (*CENPB*, *ERGIC1*) and pancreatic adenocarcinoma (*PPRC1*, *SLC44A1*). Eleven compounds are similar to Bruceine D, which appears to induce the down-regulation of *POMGNT1*, and is associated with improved survival in lymphoid neoplasm diffuse large B-cell lymphoma and skin cutaneous melanoma. One compound is similar to Withaferin A, which induces the down-regulation of 4 different genes (*CD47*, *CENPB*, *PAQR4*, *SLC44A1*), associated to an improved survival in uterine corpus endometrial carcinoma (*CD47*), brain lower-grade glioma (*CENPB*), kidney renal clear cell carcinoma (*PAQR4*), kidney renal papillary cell carcinoma (*PAQR4*), liver hepatocellular carcinoma (*PAQR4*) and pancreatic adenocarcinoma (SLC44A1). Six compounds are similar to Japonicone A which induces the down-regulation of *PPRC1*, which enhances the survival in pancreatic adenocarcinoma (Table S4).

**Figure 4:**
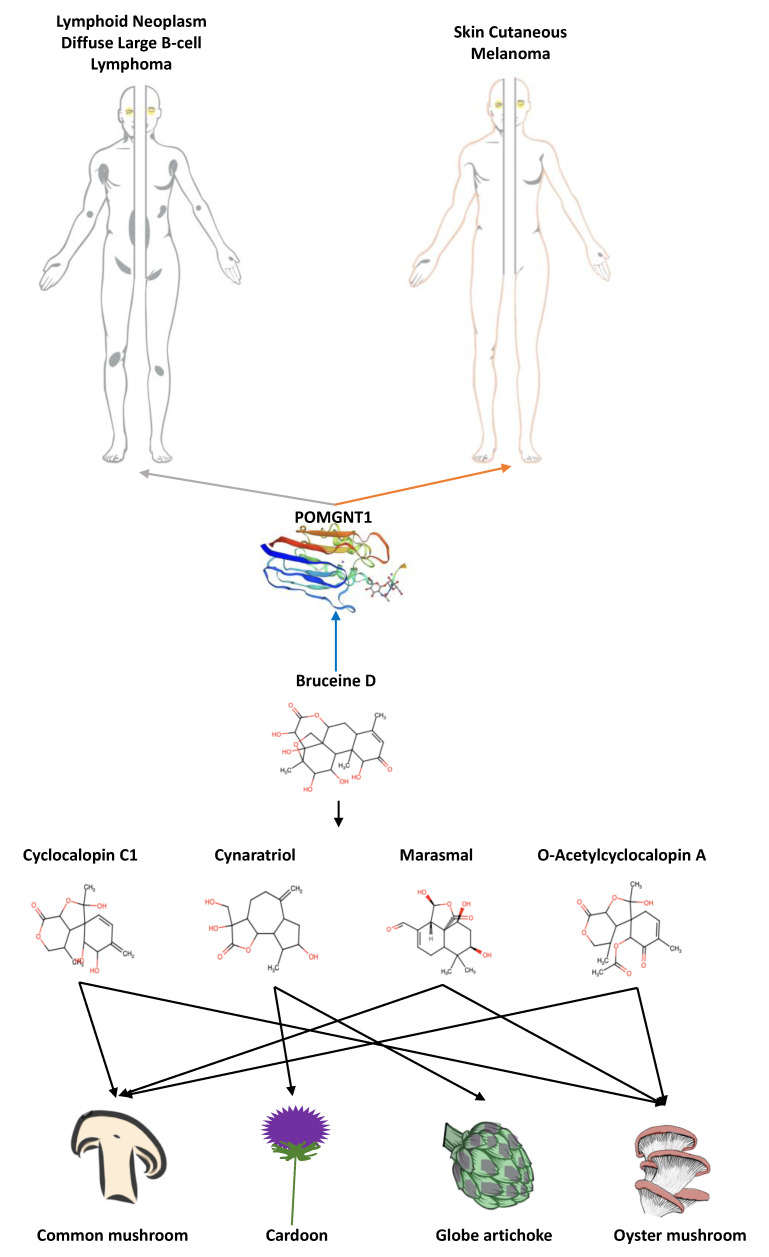
Potential novel foods useful in cancer treatments and/or prevention. Four new identified compounds that share similarity with Bruceine D and that could play a role in the regulation of genes that enhance the survival in lymphoid neoplasm diffuse large B-cell lymphoma and in skin cutaneous melanoma. O-Acetylcyclocalopin A, Marasmal and Cyclocalopin C1 are predicted in Oyster mushroom and in common mushroom. Cynaratriol is expected in cardoon and globe artichoke.

Considering the opportunity that the investigated compounds could act favoring apoptosis and improving survival in specific cancers, thus having a potential role in anti-cancer treatments, we searched for the presence of the 23 bioactive compounds in food, using FooDB (http://www.foodb.ca, accessed on 13 May 2021). Our analysis confirmed the presence in 4 foods for only 4 compounds, all similar in structure with Bruceine D (Figure 4).

## 4. Discussion

### 4.1. Novel Prognostic Genes in Different Cancer Types

We recently identified 734 genes that resulted co-expressed with genes involved in programmed cell death in 9 apoptotic treatments [26]. They were not yet reported as involved in apoptosis by GO and/or KEGG. Among the 149 genes that showed the same expression trend in the apoptotic experiments and in 15 nutrigenomics treatments inducing apoptosis, 22 were found to respect the conditions of having an opposite trend in at least one cancer type from TCGA [91]. Then, exploiting the TCGA data to compare survival plots from patients with different expression trends, we confirmed a favorable outcome when considering the apoptotic trends in gene expression of 7 genes. The downregulation of these genes in apoptotic-inducing experiments and the up-regulation in cancer types may suggest their active role in the respective diseases. Table 1, beyond showing that different genes have similar expression trends in the same treatment, also highlights the cancer types in which the trends are confirmed per each gene. These 7 genes, therefore, are here proposed as novel prognostic genes for the 8 cancer types.

### 4.2. Candidate Markers and Associated Functional Partners

Among the selected genes, *CD47* encodes for a membrane receptor that belongs to the cluster of differentiation of the immunoglobulin superfamily [65]. *CD47*, here revealed to be up-regulated in uterine corpus endometrial carcinoma (Table 1), and to be associated to an unfavorable prognosis when showing this expression trend (Figure 1), has been reported to be up-regulated also in other cancers, such as acute myeloid leukemia, breast cancer, melanoma and non-Hodgkin lymphoma [92,93,94,95]. Moreover, in 2020, Liu et al. [96] already observed the up-regulation of *CD47* in endometrial carcinoma tissues, with higher expression levels in advanced tumor tissues. The up-regulation of *CD47* is reported to enhance cell viability, to suppress apoptosis and to inhibit cell cycle arrest in endometrial carcinoma [96]. Moreover, in 2008, Tsai et al. reported that CD47 can directly bind SIRPα. SIRPα diffuses laterally on the macrophage membrane and accumulates at the phagocytic synapse to bind CD47, signaling a ‘self’ cell, inhibiting the phagocytosis by the macrophage [97]. Moreover, in 2020, Huang et al. [98] reported that the binding of CD47 with SIRPα triggers the “don’t eat me” effect, which prevents cancer cells from immune clearance [98]. Our results also confirmed the prognostic role of CD47, highlighting the possible relationships with other apoptotic-related proteins revealed by the STRING analysis, such as PTK2 and FPR2 (Figure 2). *PTK2* encodes for the focal adhesion kinase, a tyrosine kinase that is a critical regulator of adhesion and motility. Its overexpression is associated with increased metastatic potential [99]. The cytosolic protein PTK2 associates with the intracellular tails of integrins and interacts with various cytoskeletal proteins. The experimental activation of *PTK2* is sufficient for epithelial cell survival in the absence of contact with the extracellular matrix. Interestingly, *PTK2* has been shown to be overexpressed in metastatic human breast and colon cancers. Moreover, *PTK2* deletions in mice are associated with the induction of cell death and the inhibition of tumor progression [100]. Furthermore, the *PTK2* promoter contains p53 responsive elements that determine a p53-dependent gene down-regulation caused by DNA damages. Indeed, loss of p53 function in breast cancer contributes to the metastatic potential of tumors through uncontrolled *PTK2* expression [99].

FPR2 is a member of the 7 transmembrane G-protein-coupled formyl peptide receptors family, that is expressed mainly by mammalian phagocytic leukocytes and plays a role in host defense and inflammation [101]. Moreover, experimental evidence suggests that FPR2 is associated with different cancer types, such as colon cancer, melanoma and ovarian cancer [102,103,104].

*CENPB* is a gene that encodes a protein that facilitates centromere formation, termed the major centromere autoantigen B [68], since its antibodies were higher in all patient sera affected by Raynaud’s syndrome [105]. We here show that its overexpression in brain lower-grade glioma (Table 1) is associated to an unfavorable tumor prognosis (Figure 1). Interestingly, the up-regulation of this gene acquired attention also in breast [106] and in lung [107] cancers. In 2005, Atalay et al. [106] demonstrated that in 55 patients with breast cancer, the anti-CENP-B antibody had higher positivity compared to the control group (25 patients), highlighting a possible role of *CENPB* cancer type [106]. In addition, in 2008, Briasoulis et al. [107] reported the detection of anti-CENP-B antibodies as prognostic markers before the establishment of the full-blown cancer on a small-cell lung [107]. The STRING analysis here reveals (Figure 2) that CENPB interacts with TRIM21, which is involved in both cancer proliferation and in innate immunity, possibly explaining its role in autoimmune diseases such as systemic lupus erythematosus and in Sjögren’s syndrome [74]. TRIM21 may enhance cancer proliferation, or alternatively, it may increase the ubiquitination of many cancer-triggering proteins, determining their proteasomal-mediated degradation. This indicates that TRIM21 may act both in cancer proliferation and in cell apoptosis, an ambivalent role that still deserves further investigations [74]. CENPB also interacts with PARP1 (Figure 2), which is part of the poly(ADP-ribose) polymerase family implicated in Poly(ADP-Ribosyl)ation (PARylation), a post-translational modification in which PARP1 cleaves NAD+ and may transfer the ADP-ribose to asparagine, aspartic acid, glutamic acid, arginine, lysine and cysteine residues on target proteins [108,109]. PARP1 is involved in many different aspects of human cell biology [109]. Indeed, it is implicated in the cellular response to DNA damage [73], in programmed cell deaths (e.g., in parthanatos or apoptosis) [69,70,71], and in cancer [72]. PARP1 became a target in clinical oncology because PARP inhibitors were identified as chemosensitizers in combination with classical DNA-damaging therapies or as mono-therapeutic agents to treat cancers [72]. In 2005, two efforts [110,111] highlighted the synthetic lethal interaction between PARP1 inhibition and loss of BRCA1 or BRCA2, which lead to the development of approved clinical PARP inhibitors in ovarian, breast or pancreatic cancer treatments in case of *BRCA1* or *BRCA2* loss-of-function. Functional BRCA1 and BRCA2 are of critical importance for the repair of double strand breaks via homologous recombination [72].

*ERGIC1* encodes for a protein involved in the transport between endoplasmic reticulum and the Golgi apparatus [75]. We here highlighted that its up-regulation in brain lower-grade glioma (Table 1) is associated to an unfavorable prognosis (Figure 1). Interestingly, high expression levels of *ERGIC1* are revealed in prostate cancer tissues, and its silencing down-regulates the *ERG* oncogene [112]. The STRING analysis here reveals (Figure 2) that ERGIC1 interacts with ARRDC3, an arrestin-related domain-containing protein that has been reported to promote lysosome-mediated protein degradation [113]. Interestingly, ARRDC3 has been reported to act as tumor suppressor in different types of cancers, such as breast, colorectal and prostate cancer [113,114,115,116,117]. Moreover, ERGIC1 interacts with HIGD1A, which is essential for mitochondrial homeostasis. In fact, *HIGD1A* knockdown resulted in mitochondrial fission, severe loss of mitochondrial DNA, disorganization of cristae and cell growth retardation [118].

The gene *PAQR4* encodes for a progestin and adipoQ receptor [78]. Here we report that the up-regulation of *PAQR4* in kidney renal clear, kidney renal papillary cell cancers and liver hepatocellular carcinoma (Table 1) is associated to an unfavorable prognosis (Figure 1). Interestingly, the up-regulation of *PAQR4* was revealed to have a tumorigenic effect also in breast cancer [119,120], lung cancer [121], and is also reported to be associated with poor survival outcome in prostate cancer [122]. The STRING analysis here reported (Figure 2) reveals that PARQ4 interacts with FLYWCH1, a transcription modulator with an FLYWCH/Zn-finger DNA-binding domain [80]. In addition, FLYWCH1 is reported to directly bind the nuclear β-catenin, efficiently suppressing the transcriptional activity of the β-catenin signaling, selectively blocking the expression of specific downstream genes associated with cell migration and morphology [80]. Moreover, PAQR4 interacts with ASB2, an ankyrin repeat-containing protein with a suppressor of cytokine signaling box-2 [79]. Upon treatment with retinoic acid, which has a force de novo differentiation in leukemia cells, *ASB2* is up-regulated and induces growth inhibition and chromatin condensation [79].

The gene *POMGNT1* encodes for an O-mannose beta-1,2-*N*-acetylglucosaminyltransferase, which participates in O-mannosyl glycan synthesis [81,82]. Here we report its up-regulation in lymphoid neoplasm diffuse large B-cell lymphoma and in skin cutaneous melanoma (Table 1), which is associated to an unfavorable prognosis in both cancers (Figure 1). Interestingly, *POMGNT1* is reported to be up-regulated in glioblastoma apoptosis resistant cell lines, whereas the *POMGNT1* knock-down enhanced apoptosis when cells are treated with temozolomide [123]. The STRING analysis here performed reveals that POMGNT1 interacts with DAG1 (Figure 2). *DAG1* encodes for the dystroglycan complex, which is composed by the α and the β subunits, identified in skeletal muscle and brain [124] and involved in dystrophic phenotypes [84]. The dystroglycan locates at the interface between the cell membrane and the basement membrane. It forms a continuous link from the extracellular matrix to the intracellular actin cytoskeleton, providing structural integrity and perhaps transducing signals, resembling the integrin role. Thus, loss of the dystroglycan expression might perturb the interactions between cells and the surrounding environment, and might contribute to the deregulation of the cell ability to interact with basement membrane and/or with the surrounding extracellular matrix—events that are frequently observed in the development and progression of many types of cancer. Indeed, defects in extracellular matrix organization and perturbations of basement membrane organization in the epithelial and stromal compartments are considered a hallmark of malignant transformation [83].

The gene *PPRC1* encodes for a peroxisome proliferator-activated receptor γ coactivator 1, which is linked to mitochondrial biogenesis because of its ability to activate nuclear genes encoding mitochondrial proteins [85]. Here we report its up-regulation in pancreatic adenocarcinoma (Table 1) and its association to an unfavorable prognosis (Figure 1). Interestingly, Savagner et al. [125], in 2003, demonstrated the *PPRC1* up-regulation in thyroid oncocytoma [125], a tumor type that is characterized by dense mitochondrial accumulation, a phenomena that the authors commented to be a consequence of the overexpression of *PPRC1* [125]. The STRING analysis here reveals that PPRC1 interacts with TRUB1 (Figure 2), a synthase that catalyzes the pseudouridylation of mRNAs [126]. In 2020, Kurimoto et al. [127] described the essential role of TRUB1 in the maturation of the miRNA let-7, an evolutionary conserved microRNA that mediates a post-transcriptional gene silencing regulating a wide range of biological processes, including development and differentiation [127]. The authors proposed that TRUB1 acts as a suppressor of cell proliferation, and therefore as a tumor suppressor, which is mediated in part by let-7 [127]. The STRING analysis also revealed that PPRC1 interacts with NOP56 and DKC1. NOP56 is involved (with other ribonucleoproteins (RNPs) and snoRNAs) in the 2′-O-methylation of target RNAs [128], whereas DKC1 is a pseudouridine synthase, as in the case of TRUB1, that guides the pseudouridylation (with other RNPs and snoRNAs) of target RNAs [129]. In 2017, Gong et al. [130] highlighted that *NOP56* and *DKC1* were overexpressed in more than five different cancer types, in association with a general up-regulation of the total snoRNAs [130]. Moreover, they observed that NOP56 and DKC1 overexpression was associated with poor survival prognosis [130]. NOL6 is also associated in the STRING network with PPRC1. It is a nucleolar RNA-associated protein involved in pre-rRNA primary transcripts processing and in ribosome biogenesis [131]. In 2020, Dong et al. [132] identified the role of NOL6 as a human prostate cancer oncogene [132]. They showed that the *NOL6* knock-down blocks cell mitosis, decreasing cell proliferation and favoring apoptosis [132]. Finally, PPRC1 is also associated with ATP6V0C. *ATP6V0C* encodes for a vacuolar ATPase that, when silenced, determines the suppression of the migration of prostate carcinoma cells [133].

*SLC44A1* encodes for an important mediator of the choline transport across both the plasma membrane and the mitochondrial membrane [89]. Here we report its up-regulation in pancreatic adenocarcinoma (Table 1) and the association to an unfavorable prognosis (Figure 1). Interestingly, in 2015, Panett et al. [134] already reported on the role of *SLC44A1* in pancreatic ductal adenocarcinoma, proposing its role as a suitable molecular marker [134]. Our analysis confirmed this role, and the STRING analysis also reveals that SLC44A1 may interact with SH3BP5 (Figure 2), an essential kinase for B-cell differentiation and proliferation [135]. In 2019, Li et al. [90] reported that the elevated expression of *SH3BP5* significantly also correlated with poor outcomes of acute myeloid leukemia patients [90]. Through the knock-down of *SH3BP5*, the authors demonstrated the inhibition of cell viability and the induction of apoptosis [90].

In summary, our analysis confirmed the role of 2 genes (*CD47* and *SCL44A1*) in 2 cancer types (uterine corpus endometrial carcinoma and pancreatic adenocarcinoma, respectively) and highlighted 5 novel marker candidates in 6 cancer types (Figure 3).

### 4.3. Putative Effectors from Nutritional Treatments

Natural products and their structural analogues have historically made a major contribution to pharmacotherapy, especially for cancer, bringing great interest in natural compounds as drug leads [136]. Computational approaches sustain drug discovery [137], performing virtual screening approaches for searching molecules that are similar to known active compounds [30], and predicting the pharmacokinetics, drug likeness and medicinal chemistry friendliness of newly identified molecules [31].

Starting from the 6 compounds exploited in the nutrigenomics treatments here selected for their role in inducing the down-regulation of the 7 genes that showed higher expression in specific cancers (Table 1), we investigated on additional similar compounds that could exert the same effects [30]. Excluding the rosemary extract, since the molecular composition for this treatment is not clearly defined, we identified 231 compounds (Table S3). Predicting their ADME [31], we selected 23 bioactive compounds that respect Lipinski [57], Ghose [58], Veber [59], Egan [60] and Muegge [61] rules (0 violation); Pan Assay Interference Compounds [62], Brenk [63] and lead likeness [64] (≤1 violation), and with high gastrointestinal absorption (Table S4). All these compounds are to be considered suitable candidates for further tests in at least the cancer types here described (Figure 3). Successful outcomes from these tests could pave the way to novel opportunities for cancer treatments or for the design of functional foods.

Among the 23 additional bioactive compounds, it is interesting to mention some compounds that are already known to be effective in cancer treatments.

Triptolide, a terpene [50], shares similarity with Bruceine D (at 61.7% similarity) (Table S3). Interestingly, Triptolide is already known to induce apoptosis in promyelocytic leukemia, in T-cell lymphoma [138] and in melanoma [139].

Tubocapsanolide A, an alcohol [50], shares a 98% similarity with Withaferin A (Table S3), which, in our analysis, appeared to enhance the survival in brain lower-grade glioma, uterine corpus endometrial carcinoma, kidney renal clear cell carcinoma, kidney renal papillary cell carcinoma and pancreatic adenocarcinoma (Figure 3, Table 1). Interestingly, Tubocapsanolide A is independently reported to inhibit proliferation of human lung cancer [140].

Cordysinin C and D share 88.7% and 84.4% similarity with Indole-3-carbinol, respectively (Table S3). These two compounds are known to have anti-inflammatory properties [141], while here we report that they could have activity against uterine corpus endometrial carcinoma, brain lower-grade glioma and pancreatic adenocarcinoma (Figure 3).

### 4.4. Opportunities from Functional Foods in Cancer Treatments

Nowadays, industry and research are highly focused on health and wellness. In fact, worldwide sales of naturally healthy foods reached $253 billion in 2017 whereas functional foods totaled $247 billion [142]. Functional foods can act as anti-cancer agents [143]; in fact, several large randomized clinical trials are ongoing to clarify the impacts of functional foods on cancer management and prevention [144]. Functional foods do not cure or prevent illnesses by themselves but must be viewed in the context of a healthy diet to exert their potential interest.

To move a step forward towards the identification of nutrients or foods that could be revealed to be active in cancer prevention and/or treatment, we performed a preliminary investigation on foods that contain or that are predicted to contain some of the 23 compounds here selected. We point out 4 different foods, having 4 out of the 23 bioactive compounds (Figure 4). All these 4 compounds share similarity with Bruceine D, thus revealing possible candidate foods to be considered for a possible role in lymphoid neoplasm diffuse large B-cell lymphoma and in skin cutaneous melanoma.

Among these 4 compounds, O-Acetylcyclocalopin A, Marasmal and Cyclocalopin C1 are predicted to be compounds present in *Pleurotus ostreatus* (Oyster mushroom) and in *Agaricus bisporus* (common mushroom). Noticeably, Oyster mushroom is already known to have an anti-tumoral activity against lymphoma and melanoma [145,146], whereas the common mushroom shows an anti-tumoral activity against melanoma [147]. Finally, Cynaratriol is expected but not yet quantified in *Cynara cardunculus* (cardoon) and *Cynara scolymus* (globe artichoke). Although they are known for their beneficial effects for human health [148], here, for the first time, cardoon and globe artichoke are suggested to be suitable candidates for their anti-tumor role against lymphoma and/or melanoma.

## 5. Conclusions

In this study, we employed a computational strategy to appropriately exploit bioinformatics data resources and tools to investigate on novel gene candidates involved in apoptosis, and affected in their expression by compounds identified by nutrigenomics experiments. This effort resulted in 7 novel genes that have been shown to be down-regulated in apoptosis and in nutrigenomics treatments inducing programmed cell death and up-regulated with an associated unfavorable outcome in 8 cancer types. Among these 7 genes, 5 can be considered novel prognostic candidate markers for the cancer types here considered as well as suitable targets of dedicated molecular investigations for their roles and their interaction with proteins involved in peculiar cell functionalities. On the other hand, a couple of them finds here an independent confirmation, since they are already considered in cancer treatments. In addition, we selected 6 compounds from nutrigenomics treatments and 23 bioactive compounds similar to 5 of them that responded to ADME (adsorption, distribution, metabolism and excretion of small molecules) prerequisites for being suitable candidates for translational applications.

Finally, a preliminary research for potential functional foods resulted in 4 unprocessed foods containing 4 among the 23 bioactive compounds, all of them similar to Bruceine D, potentially useful against non-Hodgkin lymphoma and skin melanoma.

A validation of the candidate marker genes here detected, of the effective bioactivity of the selected compounds in treating at least those cancers here highlighted to be associated to the unfavorable up-regulation of these genes, are mandatory to translate these results into practice. Nevertheless, our results highlight also the relevance of public data resources such as the ones here exploited, since they can strongly impact this field of research. The precious opportunity to move from gene expression data to candidate compounds for cancer treatment and prevention, favored by computer-aided selections of potential useful molecules, paves a faster way to novel drugs and functional food design.

## Figures and Tables

**Table 1:** List of the 7 genes with confirmation of enhanced survival rate in cancer types and expression patterns in apoptotic, nutrigenomic and cancer.

	Treatments										Cancers							
	Apoptotic		Nutrigenomic															
	TMX	Al-10-49	Rosemary	Withaferin A	Bruceine D	Japonicone A	Indole3carbinol	Indole3carbinol	Indole3carbinol	Eusynstyelamide B	DLBC	KIRC	KIRP	LGG	LIHC	PAAD	SKCM	UCEC
Cell line	MCF7	ME1	SW620	MDA	MCF7	MCF7	MCF7	T47D	ZR75	LNCaP								
Gene Symbol																		
CENPB	-	−1.3	-	−1.5	-	-	−1.5	-	-	-	Up	-	-	Up/*	-	Up	-	-
ERGIC1	−1.2	-	-	-	-	-	-	−1.7	-	-	-	Up	-	Up/*	-	Up	-	-
CD47	−1.3	-	-	−2.1	-	-	−1.6	−1.8	−1.9	-	Up	-	-	-	-	Up	-	Up/*
PAQR4	−1.5	−2.3	−3.1	−1.8	-	-	-	-	-	−1.9	Up	Up/*	Up/*	-	Up/*	Up	Up	Up
POMGNT1	-	−1.1	-	-	−1.6	-	-	-	-	-	Up/*	-	-	-	-	Up	Up/*	-
PPRC1	-	−1.9	-	-	-	−1.5	−2.1	-	-	-	Up	-	-	-	-	Up/*	-	-
SLC44A1	−1.1	-	-	−1.8	-	-	-	−1.9	-	-	Up	-	-	Up	-	Up/*	-	-

Gene identifier (Gene Symbol), expression pattern (log2 fold change) in apoptotic (TMX and Al-10-49) and nutrigenomic treatments (rosemary, Withaferin A, Bruceine D, Japonicone A, Indole3carbinol and Eusynstyelamide B), as well as treated cell lines are shown. For TCGA cancer types, gene regulation is reported, too (UP and DOWN). *: Cells with asterisks indicate TCGA cancer types in which the gene expression pattern in the apoptotic conditions is associated to a favorable survival outcome (Figure 1). Note: DLBC: lymphoid neoplasm diffuse large B-cell lymphoma, KIRC: kidney renal clear cell carcinoma, KIRP: kidney renal papillary cell carcinoma, LGG: brain lower-grade glioma, LIHC: liver hepatocellular carcinoma, PAAD: pancreatic adenocarcinoma, SKCM: skin cutaneous melanoma, UCEC: uterine corpus endometrial carcinoma.

## Data Availability

Publicly available datasets were analyzed in this study. Data source and accessed date are described in materials and methods. Data are also available in Supplementary Materials.

## Supplementary Materials

The following data are available online at https://www.mdpi.com/article/10.3390/cancers13164207/s1, Table S1: List of the 149 genes and their expression patterns in 9 apoptotic treatments [26] and in 15 NutriGenomeDB experiments inducing apoptosis, Table S2: List of the 22 genes showing opposite expression patterns in apoptosis and in cancer types, Table S3: The list of 231 molecules that are similar to the reference compounds identified by nutrigenomic treatments, Table S4: List of the AMDE parameters for the 23 compounds.
